# Achieving 1.7 GPa Considerable Ductility High-Strength Low-Alloy Steel Using Hot-Rolling and Tempering Processes

**DOI:** 10.3390/ma17184495

**Published:** 2024-09-13

**Authors:** Haoyu Geng, Xiangyu Sun, Xingsen Guo, Yajun Zhao, Xingjie Yin, Zhiming Du

**Affiliations:** 1 School of Materials Science and Engineering, Harbin Institute of Technology, Harbin 150001, China; genghy001@163.com (H.G.); sunxiangyu1166@163.com (X.S.); tian17852227221@163.com (Y.Z.); 17852229665@163.com (X.Y.); 2 Zhangjiakou Sanxin Tongda Machinery Manufacturing Co., Ltd., Zhangjiakou 076250, China; guoxs118@sina.com

**Keywords:** high-strength low-alloy steel, hot rolling, tempering, martensitic transformation, strengthening mechanism

## Abstract

To achieve a balanced combination of high strength and high plasticity in high-strength low-alloy (HSLA) steel through a hot-rolling process, post-heat treatment is essential. The effects of post-roll air cooling and oil quenching and subsequent tempering treatment on the microstructure and mechanical properties of HSLA steels were investigated, and the relevant strengthening and toughening mechanisms were analyzed. The microstructure after hot rolling consists of fine martensite and/or bainite with a high density of internal dislocations and lattice defects. Grain boundary strengthening and dislocation strengthening are the main strengthening mechanisms. After tempering, the specimens’ microstructures are dominated by tempered martensite, with fine carbides precipitated inside. The oil-quenched and tempered specimens exhibit tempering performance, with a yield strength (YS) of 1410.5 MPa, an ultimate tensile strength (UTS) of 1758.6 MPa, and an elongation of 15.02%, which realizes the optimization of the comprehensive performance of HSLA steel.

## 1. Introduction

HSLA steel has been widely used in engineering due to its excellent performance, and it has always been a popular research topic. HSLA steel has a low alloy content, ultrahigh strength, excellent ductility, high effective cost, and good weldability. This steel is widely used in various fields, such as bridges, high-rise buildings, oil and gas pipelines, mining machines, ships, and marine platforms, effectively supporting human economic activities [1,2,3]. With the development of modern industry and economy and the emergence of issues with resources and the environment, the performance characteristics of low-alloy steels have faced further challenges. The development of high-performance low-alloy steels that exhibit high strength, plasticity, toughness, wear resistance, and good welding performance has become inevitable.

In the process of HSLA steel being prepared by a conventional smelting process, the higher melting temperature leads to the volatilization of the lower-melting-point elements and internal defects such as elemental segregation and porosity. The lower sintering temperature of the powder metallurgy process (PM) can effectively mitigate compositional segregation and coarse grains formation in traditional casting processes. It is easy to obtain composites of many different materials and fully exploit the properties of each material, attracting increasing attention from researchers [4,5,6]. For example, Chen et al. [7] introduced vanadium nitride (VN) particles into tungsten–molybdenum high-speed steel (M2 steel) using powder metallurgy, where VN particles were uniformly distributed in the matrix, and the grain size was refined to 0.7 μm, with no coarse grains found. Unfortunately, powder metallurgy materials are partially porous internally and often do not become fully dense, thereby affecting the mechanical properties of HSLA steels. The elimination of sintered pores by a hot-rolling process after sintering is an excellent solution. Duan et al. [5] rolled 30CrMnSiNi2A steel obtained by hot isostatic pressing sintering at 1000 °C, successfully eliminating sintering pores and achieving an excellent strength–toughness balance in 30CrMnSiNi2A steel.

Suitable heat treatments can be applied to effectively regulate the microstructure, which is essential for achieving materials with high strength and toughness properties [8,9,10,11]. Varying parameters during heat treatment can significantly change the microstructures and properties due to austenite decomposition. Quenching and tempering treatment, or normalizing and tempering treatment after complete austenitization, produces martensite/bainite structures and improves the mechanical strength of HSLA steel [12,13]. The parameters of heat treatment are critical for achieving an optimum balance between toughness and high strength. The austenitizing temperature essentially contributes to the prior austenite grain (PAG) size [14,15]; furthermore, controlling the austenitizing temperature can promote the formation of grain refinement structures. In addition, other parameters, such as the cooling rate and final cooling temperature after heat treatment, significantly impact the microstructures of steels [16,17], and regulating these parameters can affect the mechanical properties of these materials to accommodate specific applications. Many researchers have studied martensitic transformation [18,19,20]. However, martensite is a complex and brittle phase that sacrifices ductility to improve strength. The trade-off between high strength and high ductility is seemingly a significant issue in developing HSLA steels. Therefore, an appropriate heat treatment process is essential for developing a new generation of HSLA steels with excellent combined properties. Microalloying, controlled rolling processes, heat treatment processes, and other methods are commonly used to adjust the comprehensive mechanical properties of HSLA steels, but fewer studies have been conducted on improving the mechanical properties of HSLA steels through the combined processes of powder metallurgy and rolling.

Due to these considerations, we use a new process of powder metallurgy combined with hot rolling and tempering to prepare PM low-alloy high-strength steel for detailed study of the effects of post-rolling air cooling, oil quenching, and subsequent tempering treatments on the microstructure and mechanical properties of HSLA steel, and analysis of the toughening mechanism of HSLA steel. The main purpose of this paper is to investigate the effect of different cooling conditions and medium-temperature tempering after rolling on austenite phase transformation and carbide precipitation to prepare high performance powder metallurgy HSLA steel. It provides a reference for the development of high-strength and high-elongation HSLA steel. Subsequently, we will further regulate the microstructure and mechanical properties according to tempering temperature and tempering time.

## 2. Materials and Methods

The studied steel had a chemical composition of 0.18C, 0.60Si, 1.36Mn, 1.40Cr, 0.20Mo, and 1.00Ni (wt.%). The mechanical alloying (MA) process was lubricated by adding 0.6 wt.% zinc stearate with a ball–material ratio of 5:1. The ball milling speed was 300 rpm, and grinding was stopped every 60 min for 10 min to prevent excessive welding caused by the high ball milling speed. The total ball milling time was 24 h. The evenly mixed powder was pressed into a Φ60-mm-diameter block with a compression pressure of 800 MPa. The specimens were heated to 1250 °C using a chamber vacuum furnace at a rate of 5 °C/min, held for 60 min, and cooled in a furnace.

**Figure 1:**
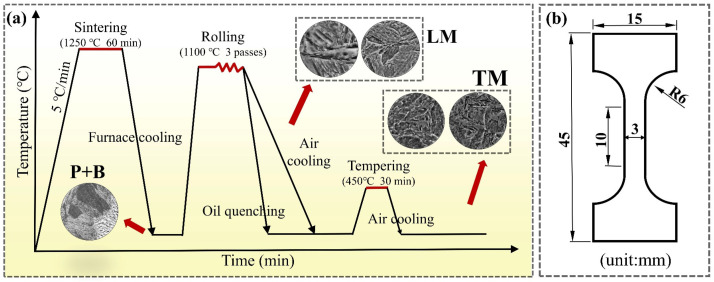
(**a**) Routes of rolling and heat treatment; (**b**) tensile sample geometry.

The sintered specimens were processed into blocks with thicknesses of 15 mm, rolled via multiple passes at 1100 °C to obtain 4-mm-thick plates, and subjected to air cooling (AC) and oil quenching (OQ) treatments. The AC and OQ specimens that were tempered at 450 °C for 30 min were called ACT and OQT specimens, respectively. Figure 1 shows the schematic diagram of the experimental steel preparation process.

The microstructure was characterized using field emission scanning electron microscopy (SEM; Gemini560, Oberkochen, Baden-Württemberg, Germany) equipped with energy dispersive X-ray spectroscopy (EDS, Oberkochen, Baden-Württemberg, Germany), electron backscatter diffraction (EBSD, Oberkochen, Baden-Württemberg, Germany), and transmission electron microscopy (TEM; Tecnai G2 F30, Hillsborough, OR, USA). The surfaces of the specimens were polished using #3000 sandpaper (Xianning, Hubei, China). Then, the specimens were subjected to polishing treatment and etched with a 10 vol% nitric acid alcohol solution. In addition, ion polishing with an energy of 5 KeV was performed on the surfaces of the specimens used for EBSD analysis. Specimens for TEM analysis were ground and polished to a thickness of approximately 50 μm and thinned with an ion-thinning apparatus. The hardnesses of the HSLA steel specimens were tested using a Vickers hardness tester under a test load of 0.98 N and a loading time of 10 s. Ten points were measured for each specimen and then averaged. The dimensions of the dog-bone-shaped tensile specimens were 10 mm × 3 mm × 1.5 mm, with the direction of the tensile axis parallel to the rolling direction, as shown in Figure 1b. The tensile performance of the HSLA steel was measured using an electronic universal testing machine at a constant speed of 1 mm/min, and the average values of three measurements were taken for the experimental results. 

Phase analysis was determined by X-ray diffraction (XRD, X’PERT) with Cu-Kα radiation. The dislocation density was measured using the Williamson–Hall method [21], and the dislocation density (*ρ*) was calculated using Equation (1) [22]; the lattice strains (*ε*) can be estimated using Equation (2). Measurement of the volume fraction of residual austenite (*V_γ_*) was determined using a modified Miller’s method.

(1) $$\rho \;=k\varepsilon^{2}/b^{2}$$

(2) βcosθ=2εsinθ

(3) $$V_{\gamma }=1.4I_{\gamma }/(I_{\alpha }+1.4I_{\gamma })$$

where *b* is the Burgers vector (0.249 nm), and k is a geometrical constant (14.4) [22]. *β* is the spectral integration line width of the diffraction peak; *θ* is the Bragg angle. *I_α_* and *I_γ_* are the integrated intensities of the (110)*α*, (200)*α*, and (211)*α* peaks and the (220)*γ* peaks, respectively. Data analysis was performed using Jade 6.0 software.

## 3. Results

### 3.1. Microstructural Characterization

**Figure 2:**
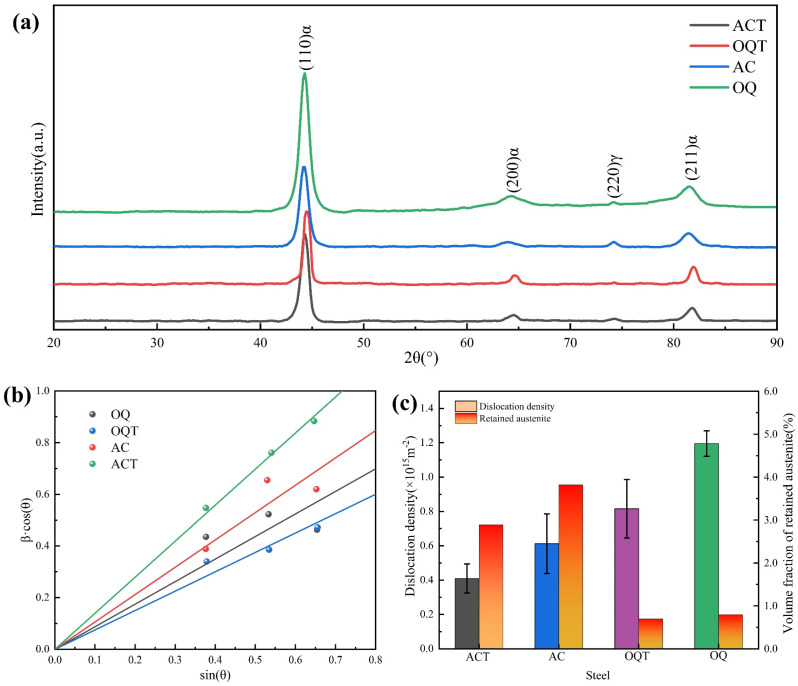
(**a**) The measured XRD patterns; (**b**) Williamson−Hall plots for those diffraction patterns of the specimens; (**c**) the volume fraction of RA and the dislocation density.

The XRD patterns and austenite contents of the four specimens are shown in Figure 2 and Table 1, respectively. Due to the limited resolution of the equipment, no carbide diffraction peaks were detected, and the microstructure of the alloy steel is mainly composed of *α* (bcc phase) and *γ* (fcc phase), as shown in Figure 2a. The XRD results illustrate the presence of residual austenite in the AC, OQ, and tempered specimens after rolling. (220)*γ* corresponds to a stronger peak intensity in the AC and OQ specimens, and the intensity of the peak decreases significantly after tempering treatment. Residual austenite was generated during the cooling process, with the highest residual austenite content in the AC specimen at 3.82%. The intensity of the diffraction peaks decreased, and the diffraction angle increased significantly after tempering. This indicates that carbon atom diffusion, phase transformation, and carbide precipitation occurred during the tempering process.

Figure 2b shows the Williamson–Hall plot obtained by the XRD patterns, and the lattice strain *ε* can be obtained by fitting the slope of the line. Figure 2c shows the dislocation density of alloy steel under different conditions. The dislocation densities of the AC, OQ, ACT, and OQT specimens were 0.44 × 1015 m^−2^, 1.12 × 1015 m^−2^, 0.32 × 1015 m^−2^, and 0.65 × 1015 m^−2^, respectively. The results indicate that the OQ has a faster cooling rate compared to the AC specimen, and a large number of dislocations internally after thermal deformation. After tempering treatment, supersaturated carbon atoms generate carbides in the matrix, reducing dislocation density.

**Figure 3:**
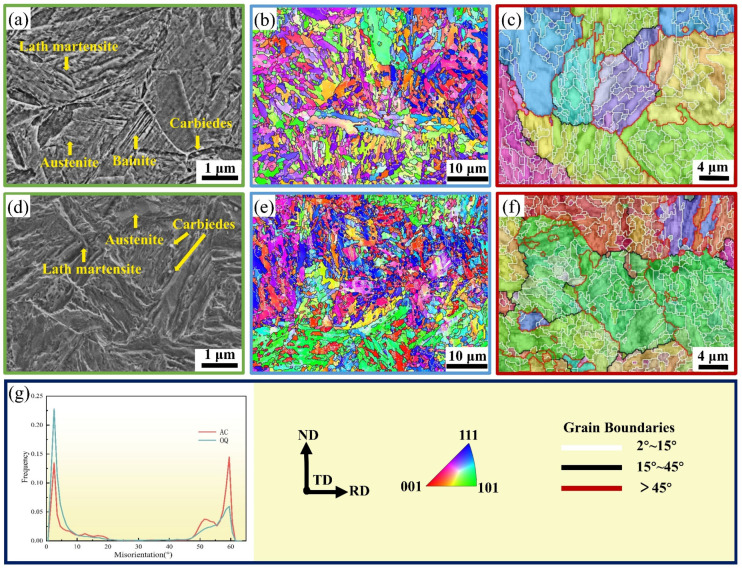
Corrosion microstructure and crystallographic characteristics of AC and OQ specimens: (**a**) corrosion SEM of AC specimen; (**b**) IPF map of AC specimen; (**c**) reconstructed PAGs and grain boundary map of AC specimen; (**d**) corrosion SEM of OQ specimen; (**e**) IPF map of OQ specimen; (**f**) reconstructed PAGs and grain boundary map of OQ specimen; (**g**) misorientation distributions of grain boundaries.

The microstructures of the AC and OQ specimens after hot rolling are shown in Figure 3. The AC specimen has a typical lath martensite (LM) structure and a small amount of bainite (Figure 3a); in contrast, the OQ specimen has a full lath martensite structure (Figure 3d). During the slow AC process, the austenite grains partially transform into bainite before the martensitic transformation occurs, dividing the austenite grain structure into relatively small regions. Thus, the bainite laths effectively refine the austenite grains [12]. The OQ specimens undergo a martensitic phase transformation during cooling and have typical lath martensite microstructures. OQ specimens have fast cooling rates, low carbon atom precipitation rates, and increased relative carbon contents; these characteristics drive martensite transformation and, ultimately, the formation of fine lath martensite [23].

The crystallographic characteristics of the AC and OQ specimens are further investigated using EBSD. The orientation differences between the martensitic laths are small, indicating no significant differences in color, whereas the martensitic block structure has significant orientation differences (>15°), representing effective grains [24]. Typical inverse pole figures (IPFs) of the AC and OQ specimens are shown in Figure 3b,e, where different colors represent different crystal orientations, the two specimens exhibit clear body-centered cubic structures, and no obvious grain orientation can be observed. The crystals become predominantly elongated in morphology, with small portions becoming large. The grain sizes of the AC and OQ specimens are significantly refined from the sintered state, with average grain sizes of 1.61 μm for the AC specimen and 1.45 μm for the OQ specimen. Hot rolling can significantly refine the grain size, which is a reason why steel requires rolling treatment. Figure 3c,f shows the reconstruction of PAGs and the grain boundaries of the AC and OQ specimens, and a statistical analysis is performed on the grain boundary dispersion (Figure 3g). The prior austenite undergoes intense plastic deformation at high temperatures, and it has irregular PAGs, indicating that the recrystallization process is inadequate. The grain boundaries of the prior austenite and lath microstructure have large misorientations, effectively inhibiting crack extension [25]. The phase transformation products of the AC and OQ specimens conform to the K–S orientation relationship with the of austenite grains [5].

In the grain boundary distribution map, 2–15° represents low-angle grain boundaries (LAGBs, white line), 15–45° represents medium-angle grain boundaries (MAGBs, black line), and >45° represents high-angle grain boundaries (HAGBs, red line). From Figure 3g, it can be seen that the grain boundaries’ misorientations trends are similar for the AC and OQ specimens. The grain boundary orientations between the AC and OQ specimens mainly deviate in two ranges: LAGBs and HAGBs. The proportions of grain boundaries with orientation deviations of 15~45° are the same. In the AC specimens, the proportions of HAGBs with orientation deviations greater than 45° are relatively high, suggesting that the effective grains of the HAGB cladding are refined. It has been shown that materials with high HAGB percentages have excellent elongation and impact toughness due to the deflecting and hindering effect of high-angle grain boundaries on microcrack extension [26,27].

**Figure 4:**
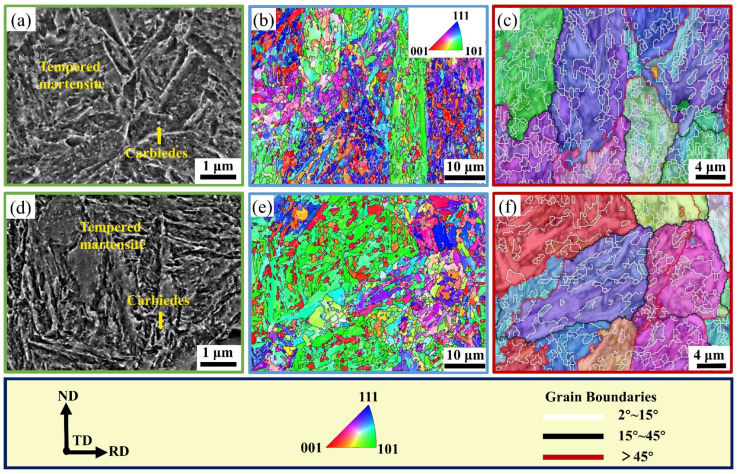
Corrosion microstructure and crystallographic characteristics of ACT and OQT specimens: (**a**) corrosion SEM of ACT specimen; (**b**) IPF map of ACT specimen; (**c**) reconstructed PAGs and grain boundary map of ACT specimen; (**d**) corrosion SEM of OQT specimen; (**e**) IPF map of OQT specimen; (**f**) reconstructed PAGs and grain boundary map of OQT specimen.

The overall morphologies of the ACT and OQT specimens change only slightly compared to those of the hot-rolled specimens, and the lath morphologies are blurred. The lath-shaped bainite is distributed in a cluster of martensitic laths, leading to the segmentation and refinement of the martensitic laths. The dimensions of the original austenitic grains, martensitic lath groups, lath bundles, and laths remain essentially unchanged. The carbides that precipitate during tempering are distributed in the matrix, as shown in Figure 4a,d. The crystallographic characteristics of the ACT and OQT specimens are shown in Figure 4. The austenite grains of the tempered specimens are equiaxed with a size of approximately 20 μm. The tempered specimens exhibit lath martensitic microstructures with no apparent crystals. Lath bainite refines martensite through the segmentation of the lath bundle (Figure 4b).

**Figure 5:**
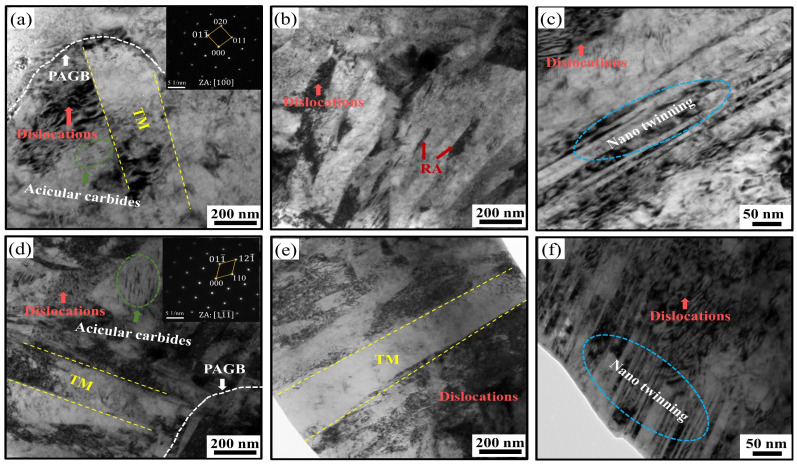
TEM images of ACT and OQT specimens: (**a**–**c**) ACT specimen; (**d**–**f**) OQT specimen.

Figure 5 shows the TEM images of the ACT and OQT specimens. Figure 5a,d shows that the microstructures of the ACT and OQT specimens consist of lath-tempered martensite (the corresponding selective area electron diffraction (SAED) pattern is marked) and film-retained austenite, with martensite lath widths of approximately 200–300 nm, a high density of dislocation structures within the tempered martensite, the presence of a twinned microstructure, and a reduction in the number of lattice distortions in the martensite laths, which leads to the further release of internal stresses [23]. The lath-like tempered martensite microstructure grows along the PAG boundaries (PAGBs), which agrees with the findings of other studies [16]. When tempered at 450 °C, the lath-tempered martensite contains nanoscale precipitates with wave morphologies; these precipitates comprise the θ-carbide phase. As shown by the green circles in Figure 5d, this phenomenon is consistent with their evolution from transitional η-carbides [28,29,30]. The carbides have a considerable mismatch with the martensitic lattice, which provides Orowan strengthening to the material. The dislocation density of the OQT specimen is greater than that of the ACT specimen. Due to the high dislocation density, additional nucleation sites are available for the precipitation of carbides during subsequent tempering, and the twinning microstructure is relatively fine (Figure 5c,f). The twinning microstructure of the ACT specimen is relatively coarse, which reduces the effective slip system and increases the brittleness.

### 3.2. Mechanical Properties

**Figure 6:**
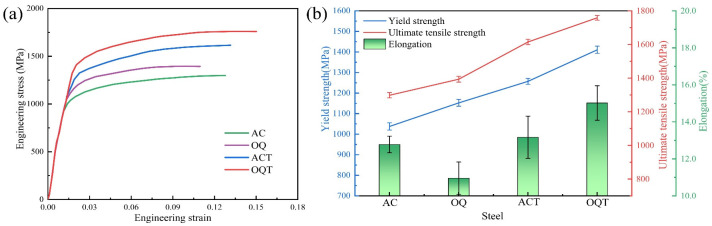
Mechanical properties: (**a**) engineering stress-strain curves; (**b**) tensile performance statistics curves.

Figure 6a,b and Table 2 show the variation in mechanical properties of different specimens. The AC specimen had a high elongation (12.78%), while the OQ specimen had a high yield strength (1152.4 MPa) and ultimate tensile strength (1393.6 MPa). After hot rolling, the internal pores were welded together, the significant oxides were broken, and the microstructure was close to the fully dense state, eliminating the defects generated during sintering. The AC and OQ specimens had martensite microstructures with high hardnesses and strengths, in addition to small amounts of bainite. Moreover, the microstructure was refined, which improved the strengthening of the grain boundaries. The elongation of the AC and OQ specimens was very limited due to the higher quench stresses and untempered lath martensite. After tempering at 450 °C, the ultimate tensile strength of the ACT specimen reached 1614.9 MPa, and the elongation decreased to 13.17%. The OQT specimen had a good combination of strength and plasticity, with an ultimate tensile strength of 1758.6 MPa, a yield strength of 1410.5 MPa, and an elongation of 15.02%. This improvement is attributed to the adjustment of residual austenite decomposition and carbide precipitation.

**Figure 7:**
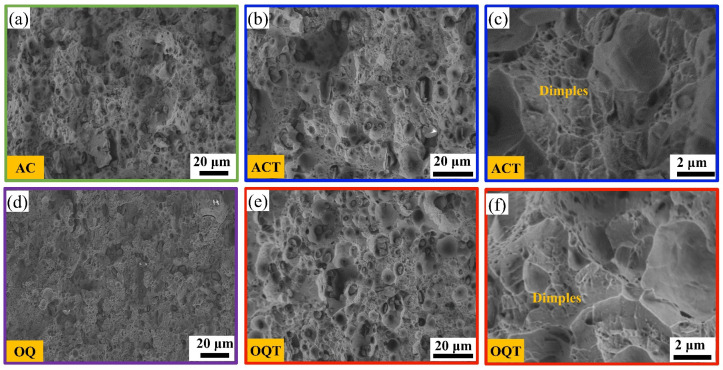
Typical fracture morphology of different specimens: (**a**) AC; (**b**,**c**) ACT; (**d**) OQ; (**e**,**f**) OQT.

The fracture morphology after stretching is shown in Figure 7. There were numerous ductile dimples inside the AC, OQ, ACT, and OQT specimens, and no dissociation steps were observed, indicating that ductile fracture was the primary fracture mechanism during tensile deformation. In addition, rolling eliminated some of the sintering defects. This elimination contributed to the improved performance. Compared with the AC and OQ specimens, the ACT and OQT specimens had more deep dimples (Figure 7b,e), indicating significant increases in the ductilities of the tempered specimens. The presence of dislocations and twins in the bainite and martensite led to significant lattice distortions, in addition to the presence of orientation differences within the lath martensite, which impeded the expansion of microcracks and improved the resistance to deformation [12].

## 4. Discussion

### 4.1. Phase Transformation Behavior during Quenching Process

HSLA steel reheats to the austenite temperature during the holding and hot-rolling stages at 1100 °C. The alloying elements are continuously enriched, and the austenite coarsens during holding. Simultaneously, rolling intensifies lattice distortion, increases dislocation density, and promotes austenite transformation [31]. The cooling rate of the AC specimen is relatively slow. When the temperature of the specimen approaches the bainite transformation point, bainite transformation begins, and the amount of bainite that undergoes transformation is relatively small. As the temperature of the specimen decreases, the austenite transforms into martensite with a high martensitic carbon content. In addition, carbon from some primary martensite diffuses into the untransformed austenite. The remaining austenite transforms into secondary martensite with increasing carbon concentration (Figure 3a). During the tempering stage, the precipitation of saturated carbon atoms in the martensite promotes the formation of carbides, and the number of carbides in the secondary martensite is much greater than that in the primary martensite [15,32]. For the OQ specimen, austenite is quickly reduced to room temperature, austenite is transformed into martensite (Figure 3d), and supersaturated carbon atoms precipitate during tempering. S.W. Thompson studied the microstructural and crystallographic characteristics of fine-scale transition carbides that form in martensite during tempering [33]. Therefore, there is a small amount of bainite in the AC specimen, and the OQ specimen has a full flat-noodles martensite structure, and fine carbides are generated in the martensite after tempering treatment (Figure 5).

**Figure 8:**
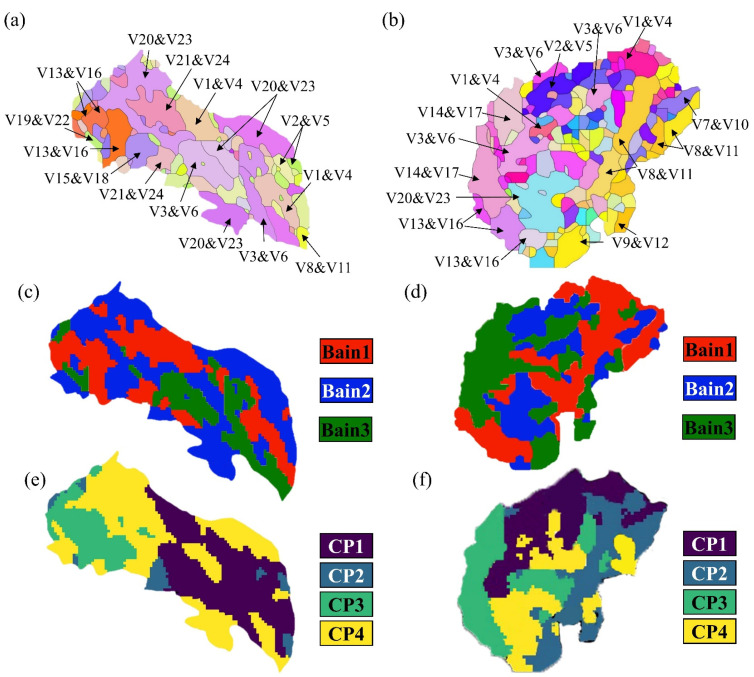
Variants distribution, CP group, and Bain group maps depicting the microstructure of representative grains in the steels: (**a**) variants distribution figure of AC specimen; (**b**) variants distribution figure of OQ specimen; (**c**) Bain group map of AC specimen; (**d**) Bain group map of OQ specimen; (**e**) CP group map of AC specimen; (**f**) CP group map of OQ specimen.

The crystallographic characteristics of the martensite were examined to reveal the transformation of the martensite, and the results are shown in Figure 8. Twenty-four variants do not necessarily appear in the austenitic grains, which is in agreement with the results of previous studies [34,35]. There are 18 variants of the AC detected in the prior austenite grains, of which six variants are missing, namely, V7, V10, V9, V12, V14, and V17 (Figure 8a). During cooling, V3, V6, V20, and V23 undergo transformation through the prior austenite grains. In the OQ specimen, six variants are missing, namely, V15, V18, V19, V22, V21, and V24 (Figure 8b). The close-packed plane (CP) and Bain maps are highlighted with four colors and three colors, respectively, as shown in Figure 8c–f. Three variants of the Bain group are arranged alternately, further dividing the grains. There are four types of CP groups in the prior austenite grains. The area occupied by the four types of CP groups was calculated using Image Pro Plus 6.0. In the AC specimen, CP1 and CP4 groups dominate, accounting for 79.94%, while, in the OQ specimen, the distribution of CP groups is relatively uniform.

### 4.2. Strengthening Mechanism of HSLA Steel

Grain size, matrix phase composition, and carbon content are the main factors affecting the strength of low-alloy high-strength steels. The AC specimen is mainly composed of bainite and martensite structures, with fine grain sizes. Grain boundary strengthening plays an important role, and residual austenite transforms into martensite during tensile deformation, giving the AC specimen high strength. The dissolution of solute atoms into the martensitic matrix in the OQ specimen produced a strong solid solution strengthening effect, resulting in a significant increase in strength. The microstructure of the quenched specimens is dominated by martensitic laths with high dislocation density and abundant twin grain boundaries (see Figure 3d and Figure 5f), and these features lead to a significant dislocation strengthening effect and grain boundary strengthening effect, which also contributes to the strength increase. The effect of dislocation density and lattice distortion is weakened after the tempering treatment of the ACT and OQT specimens, and the carbides precipitated on the matrix have a precipitation strengthening effect.

The contributions of various strengthening mechanisms to the yield strengths of the AC, OQ, ACT, and OQT specimens are investigated via yield strength modeling. The yield strength of each alloy steel is determined by the sum of the lattice friction strengthening (*σ*_0_), grain boundary strengthening (*σ_HP_*), dislocation strengthening (*σ_d_*), solid solution strengthening (*σ_s_*), and precipitation strengthening (*σ_p_*):

(4) $$\sigma_{y}=\sigma_{0}+\sigma_{HP}+\sigma_{d}+\sigma_{s}+\sigma_{p}$$

The most fundamental resistance to dislocation sliding is the intrinsic resistance that arises from the need to break bonds between atoms during dislocation sliding in the lattice, called the Peierls–Nabarro stress [36]. This stress (*σ*_0_) is usually considered 45 MPa [37,38].

Grain boundaries can effectively hinder dislocation movement, and small grains can enhance strength and plasticity. According to the Hall–Petch equation, the contribution of grain boundary strengthening can be calculated using the following equation:

(5) $$\sigma_{HP}=Kd^{-1/2}$$

where K is the Hall–Petch constant (17.4 MPa mm^−1/2^ [39]), and *d* is the grain size, which is obtained from the EBSD data in Figure 4. The average grain size of the AC specimen is 1.61 μm, and that of the OQ specimen is 1.45 μm. After tempering at 450 °C for 30 min, the block size does not change [40], and the grain boundary strengthening effect is not altered. The grain boundary strengthening value for both the AC and ACT specimens is 433.6 MPa, while the grain boundary strengthening value for both the OQ and OQT specimens is 456.9 MPa.

Dislocations are reinforced due to their remote interactions, as proposed by Taylor; this reinforcement is usually described by the following equation [41]:

(6) $$\sigma_{d}=M\alpha Gb\rho^{1/2}$$

where *M* is the Taylor factor (usually 3.06), *α* is a constant (0.24), *G* is the shear modulus (80 GPa), *b* is the Burgers vector (0.249 nm) [42], and *ρ* is the dislocation density. The strength increases produced by the dislocation strengthening effects of the AC, OQ, ACT, and OQT specimens are 306.5 MPa, 489.9 MPa, 263.4 MPa, and 371.5 MPa, respectively. The OQ specimen has the highest dislocation density, producing the most significant strength increase. The dislocation density decreases after tempering, and the dislocation strengthening effect decreases.

The presence of alloying elements in HSLA steel contributes to solid solution strengthening. The solid solution strength can be measured by the following equation [8,43,44]:

(7) $$\sigma_{s}=4570C+83Si+37Mn-30Cr+11Mo+30Ni$$

where (i) is the wt.% of element i in solution. The carbon content in the interstitial solid solution of quenched martensite (0.1–0.5 wt.% C) is approximately constant at 0.015 wt.%. In addition, most of the carbon is assumed to wholly precipitate during tempering; thus, the effect of carbon in the solid solution is negligible [44]. The atomic mismatch between Cr and Fe is minor, and the crystal structure is the same (body-centered cubic). The Cr in the solid solution has a low influence on the yield strength, and this effect can be omitted [45]. The solid solution strengthening value of the AC and OQ specimens is calculated to be 195.8 MPa, and the solid solution strengthening value of the ACT and OQT specimens is calculated to be 127.2 MPa.

The contribution of precipitation strengthening can be measured using the Ashby–Orowan equation [44]:

(8) $$\sigma_{p}=0.538Gb\sqrt{V_{f}}/X\ln\left(X/2b\right)$$

where *X* and *V_f_* are the average diameter and volume fraction of the precipitated phase, respectively.

**Figure 9:**
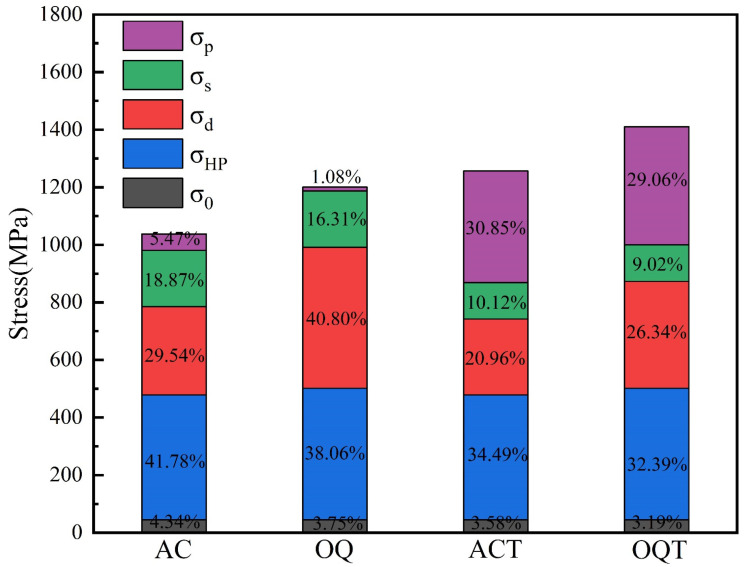
The percentage contributions of different strengthening mechanisms to yield strength.

The contributions of various strengthening mechanisms to the yield strengths of the specimens are shown in Table 3. The percentage contributions of the different strengthening mechanisms to the yield strengths are shown in Figure 9. The results indicate significant differences in the main strengthening mechanisms of the specimens before and after tempering. In the AC and OQ specimens, dislocation and grain boundary strengthening are the main strengthening mechanisms, contributing to more than 70% of the total yield strength. The contribution of precipitation strengthening after tempering treatment is enhanced, with precipitation strengthening, dislocation strengthening, and grain boundary strengthening becoming the primary contributing mechanisms. The tempering process produces needle-like carbides, which improves the effect of carbide pinning dislocations and significantly enhances precipitation strengthening. The recovery of dislocations during tempering decreases the dislocation density in the martensite, thereby reducing dislocation strengthening. In addition, tempering reduces the residual stress during quenching, thus significantly increasing elongation. Saatamoinen et al. [46] reported that during the tempering process of steel, the decrease in dislocation density corresponds to the decrease in dislocation strengthening but compensates for this with precipitation hardening and potential internal microstress elimination, resulting in the yield strength remaining unchanged or even increasing.

### 4.3. Plasticity Improvement Mechanism during Tempering

The precipitation of carbides during the tempering process keeps the material strong with strain-hardening capacity and plasticity. This section systematically investigates the plasticization mechanism during the tempering of HSLA steels.

**Figure 10:**
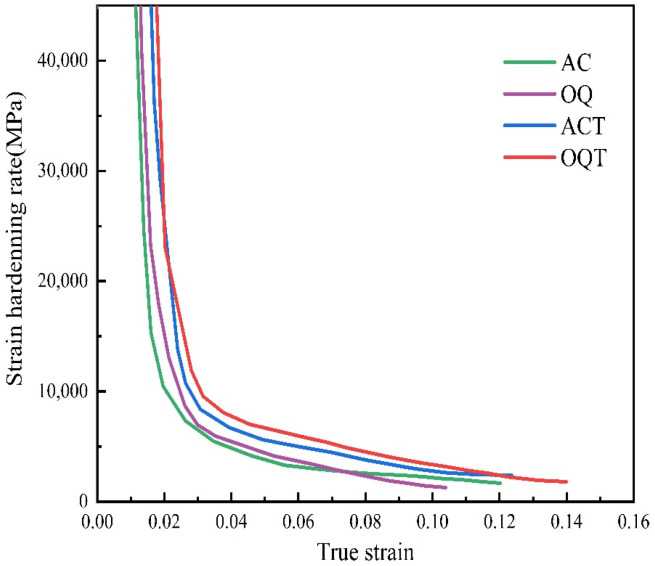
Strain-hardening rate curve.

Figure 10 shows the strain-hardening rate curves of different specimens. From the figure, it can be seen that the curve shows a sharp downward trend when the true strain increases from 0 to 0.03, which is mainly related to the dislocation slip in the microstructure during the tensile process [47]. The strain-hardening rate of the ACT and OQT specimens was greater than that of the AC and OQ specimens under the same strain condition, and the OQT specimen had the greatest strain-hardening rate and the highest work-hardening capacity. During the tempering process, residual austenite and martensite improve stability by precipitating carbides. The dislocation density in the tempered microstructure is significantly reduced (Figure 2c), leading to a decrease in its load-bearing capacity and a martensitic transformation of the residual austenite by loading during tensile processes. As a result, the work-hardening capacity of the tempered specimen is high. As the strain increases, the strain-hardening rate slowly decreases until failure. Throughout the plastic deformation process, the work-hardening rates of the ACT and OQT specimens were always greater than those of the AC and OQ specimens. The ACT and OQT specimens have great stability, and the phase-transformation-induced plastic effect can continue to occur over a large strain range, leading to the continuous growth of work-hardening. The OQT specimen maintained a long period of continuous work-hardening with a much slower monotonic decrease in work-hardening rate, ensuring a good synergy between tensile strength and elongation. This synergistic effect is related to a high martensite fraction, high-density movable dislocations, and dislocations generated by austenite-to-martensite transformation [48,49].

**Figure 11:**
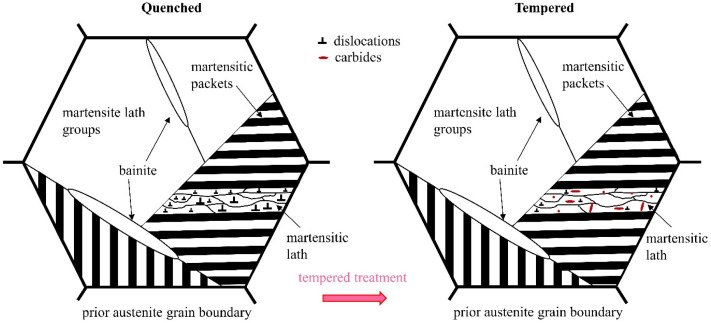
Schematic diagram of toughening mechanisms of tempered specimens.

The toughening mechanism of the tempered specimens is shown in Figure 11. The precipitation of supersaturated carbon atoms within the martensite after tempering treatment to form carbides (Figure 5a,d) leads to softening of the matrix, which increases the elongation [50]. The transformation of LAGBs into HAGBs during the tempering process leads to the deflection of cleavage cracks, which contributes to the improvement of the plasticity and toughness of the steel [51]. A large amount of internal stress is present in the AC and OQ specimens, and tempering helps to release the internal stress, thereby improving ductility. In summary, the ACT and OQT specimens have better matching strength and toughness properties.

## 5. Conclusions

In this article, the effects of hot rolling and tempering on the microstructural and mechanical properties of HSLA steel specimens are investigated. The main conclusions are as follows:

(1) The microstructure of the AC specimen consists of both bainite and martensite, with a small amount of residual austenite, and the OQ specimen has a martensitic structure accompanied by residual austenite. The AC specimen has a large number of HAGBs, which gives the material excellent elongation. Dislocation strengthening and grain boundary strengthening are the main factors for the high yield strength of the AC and OQ specimens.
(2) The microstructure of the ACT and OQT specimens consisted of tempered martensite and residual austenite, with a high density of dislocation structures within the tempered martensite, the presence of twinned martensite, and the generation of nanoscale carbides during the tempering process.
(3) The OQT specimen has good tempering resistance, with a yield strength of 1410.5 MPa, a tensile strength of 1758.6 MPa, and an elongation of 15.02%. Residual austenite and martensite during tempering improve stability by precipitating carbides. Carbide improves dislocation pinning and significantly enhances precipitation strengthening. The transition from LAGBs to HAGBs and the release of internal stress have improved ductility. The effects of precipitation strengthening, dislocation strengthening, and grain boundary strengthening are the main reasons for the high strength of the specimen.

## Figures and Tables

**Table 1:** Volume fraction of retained austenite in the different specimens calculated by XRD.

Specimen	*V_γ_* (%)	Dislocation Densities (×10^15^ m^−2^)
AC	3.82	0.44
OQ	0.80	1.12
ACT	2.89	0.32
OQT	0.68	0.65

**Table 2:** Tensile properties of different specimens.

Specimen	YS (MPa)	UTS (MPa)	EL (%)
AC	1037.7 ± 7.5	1298.2 ± 5.8	12.78 ± 0.45
OQ	1200.6 ± 6.3	1393.6 ± 7.1	10.96 ± 0.87
ACT	1257.0 ± 4.5	1614.9 ± 5.6	13.17 ± 1.14
OQT	1410.5 ± 7.7	1758.6 ± 3.9	15.02 ± 0.93

The uncertainties are quoted for a coverage factor k = (1/2).

**Table 3:** The contribution of various strengthening mechanisms to the yield strength of specimens.

Specimans	AC	OQ	ACT	OQT
*σ*_0_ (MPa)	45	45	45	45
*σ_HP_* (MPa)	433.6	456.9	433.6	456.9
*σ_d_* (MPa)	306.5	489.9	263.4	371.5
*σ_s_* (MPa)	195.8	195.8	127.2	127.2
*σ_p_* (MPa)	56.8	13.0	387.8	409.9
*σ_y_* (MPa)	1037.7	1200.6	1257.0	1410.5

## Data Availability

The original contributions presented in the study are included in the article, further inquiries can be directed to the corresponding author.
